# Legislation and Public Health

**Published:** 1898-08-13

**Authors:** 


### The Hospital

A Journal of

The Medical Sciences and Hospital Administration.

Vol. XXIV.—No. 620.] Edited by Sir Henry Burdett, K.C.B., and Solomon C. Smith, M.D., M.R.C.P. [Aug. 13, 1898.

### Legislation and Public Health.

The amount of interest which has been aroused by the action of the Government with regard to vaccination, or, rather, by the passing of the Act for the promotion of epidemics of small-pox, can hardly fail to exert a beneficial influence in directing attention to the enormous importance of the public health, and to the degree in which it is the duty of every citizen, as far as his powers and opportunities may extend, to give his vote, or to exert his influence, in favour of those candidates for Parliament or for office who will allow due weight to this potent factor in all that relates to national prosperity. It would be interesting, were it not also somewhat droll, to see the way in which gentlemen of the Tadpole and Taper class are beginning to recognise that health questions will come into greater prominence in the future than they have done in the past, and are therefore beginning, after the fashion of their kind, to seek to themselves friends among sanitary reformers. Their zeal is not always according to knowledge, and may sometimes bear a suspicious resemblance to the kind of homage which vice is proverbially said to pay to virtue; but, nevertheless, it is so far of value that it shows what, in the judgment of very keen and interested observers, is likely to become a very powerful constituent of opinion in the not very distant future. To reflections of this kind, in all probability, may be traced the somewhat ludicrous endeavour lately made in the House of Commons to attach responsibility to the Home Office, and, therefore, indirectly at least, to the Home Secretary, for the occurrence of lead poisoning in the Potteries, and of disease produced by phosphorus at the factory of Messrs. Bryant and May. Such a parade of care for the health of the "working man," or of the "working woman," would, doubtless, be read with much satisfaction in the Forest of Dean or in Berwickshire; more especially when combined with a stern determination to allow the aforesaid working man and woman to diffuse small-pox among the community without hindrance from the tyrannical interference of the State. The truth is, in relation not only to lead poisoning but to many other of the preventable diseases incidental to various forms of industry, that the employer and the Home Office are alike powerless unless assisted by the workpeople themselves; and that, for various reasons, this condition is often not at all, or only very imperfectly, fulfilled. Possibly there is in the English race a constitutional disregard of danger which entails a dislike to the trouble of precautions; possibly there is mere insensibility to the danger, or inability to realise its imminence and its character; possibly there is sometimes a fear that the occupation, whatever it may be, would bring in only a diminished wage if it were rendered safe; but certainly, whatever may be the nature of the risk incurred, or the means suggested by science or experience for diminishing it, there can be no dispute as to the fact that these means, even when liberally provided by the masters, are apt to be rendered of no account by the passive, or sometimes even by the active, opposition of the very persons for whose benefit they have been designed and furnished. Sometimes, again, the real causes of some form of suffering attributed to an industry, and affording a convenient text for the declamations of a cheap philanthropy, may be totally different from those which are commonly assigned; as in the very remarkable case to which we referred last week, in which acute lead poisoning, affecting the brain and terminating in speedy death, was not dependent upon the work performed by the patient, but upon the fact that she had taken pills containing lead in the hope of producing miscarriage. If this practice, as alleged by Dr. Crookes in his report of the case in question, be indeed prevalent among working women in the Midlands, it shows how very easily the unskilled philanthropist may be misled, and how he may come to concentrate his energies upon the extinction of an evil which has no existence except in his own imagination. What is wanted for the protection and improvement of the public health is, in the first place knowledge, especially of the real magnitude of apparent evils, and, in the second place, the appointment of a responsible Minister by whom the knowledge may be rendered available. The constitution of a Public Health Department has long been in the air, and was made a principal topic in the very interesting and instructive presidential address lately delivered by Sir Thomas Grainger Stewart to the British Medical Association. At present, however, the proposal is attended by a danger which should not be omitted from consideration. The formation of such a department is chiefly advocated by politicians of the second or third rank, who think it within the limits of possibility that they might be called upon to preside over it, and whose hopes and ambitions do not extend to the attainment of a position of high authority. Now, public health will never be duly cared for until the Minister in charge of it is of Cabinet rank, occupying the position, and wielding the powers, of a Secretary of State. For this, political opinion is not yet prepared; and it would be better to await its ripening than to begin by placing the new department in a false position of inferiority, from which it might require many years to emerge.

